# Research Advances of Non-Noble Metal Catalysts for Oxygen Evolution Reaction in Acid

**DOI:** 10.3390/ma17071637

**Published:** 2024-04-03

**Authors:** Zhenwei Yan, Shuaihui Guo, Zhaojun Tan, Lijun Wang, Gang Li, Mingqi Tang, Zaiqiang Feng, Xianjie Yuan, Yingjia Wang, Bin Cao

**Affiliations:** 1 School of Mechanical Engineering, North China University of Water Resources and Electric Power, Zhengzhou 450011, China; guo17838355546@163.com (S.G.); 13603990078@163.com (Z.T.); ligang@ncwu.edu.cn (G.L.); xjyuan@ncwu.edu.cn (X.Y.); wangyingjia@ncwu.edu.cn (Y.W.); caobin@ncwu.edu.cn (B.C.); 2 School of Materials Science and Engineering, North China University of Water Resources and Electric Power, Zhengzhou 450011, China; tangmingqi@ncwu.edu.cn (M.T.); fengzaiqiang@ncwu.edu.cn (Z.F.)

**Keywords:** non-noble metal catalysts, oxygen evolution reaction, acidic condition, activity and stability

## Abstract

Water splitting is an important way to obtain hydrogen applied in clean energy, which mainly consists of two half-reactions: hydrogen evolution reaction (HER) and oxygen evolution reaction (OER). However, the kinetics of the OER of water splitting, which occurs at the anode, is slow and inefficient, especially in acid. Currently, the main OER catalysts are still based on noble metals, such as Ir and Ru, which are the main active components. Hence, the exploration of new OER catalysts with low cost, high activity, and stability has become a key issue in the research of electrolytic water hydrogen production technology. In this paper, the reaction mechanism of OER in acid was discussed and summarized, and the main methods to improve the activity and stability of non-noble metal OER catalysts were summarized and categorized. Finally, the future prospects of OER catalysts in acid were made to provide a little reference idea for the development of advanced OER catalysts in acid in the future.

## 1. Introduction

**Figure 1:**
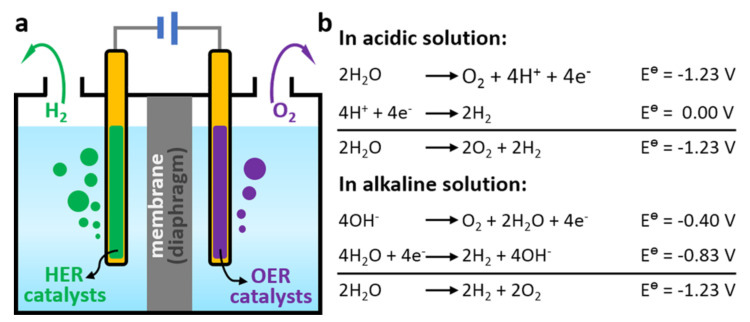
(**a**) Scheme of conventional water electrolyzers. (**b**) Water splitting reactions under acidic and alkaline conditions. Reproduced with permission. Copyright 2018, American Chemical Society [8].

Throughout history, both humans and other organisms have been deeply connected to their energy needs. Energy is fundamental to all life on Earth. In the modern era, as society and technology progress, there is a substantial consumption of energy, primarily from non-renewable fossil fuels [1,2]. Given the finite nature of these fuels and the environmental issues they pose, there is a growing emphasis on renewable energy sources [3,4]. Considering the pressing energy challenges and environmental threats, transitioning to cleaner, renewable energy alternatives to traditional fossil fuels is both essential and urgent [5]. However, renewable sources like tidal, solar, and wind power face inconsistency challenges and often fail to meet daily consumption and production demands [6]. Therefore, there is a significant push towards discovering sustainable, eco-friendly energy sources and refining their production methods. Hydrogen stands out as a viable option due to its carbon-free nature and superior energy density by weight, making it a promising clean energy medium. Electrolyzing water to produce hydrogen using electricity offers an efficient approach to renewable energy conversion [7]. In this process, water splits into oxygen and hydrogen when subjected to an electric current known as electrolysis. During electrolysis, the anode witnesses the oxygen evolution reaction (OER), while the cathode experiences the hydrogen evolution reaction (HER). Conventional water electrolysis typically occurs under acidic conditions using a proton exchange membrane (PEM) or within an alkaline setting with a separator, as illustrated in Figure 1 [8].

Under standard conditions, an electrolytic cell in an acidic medium demonstrates a current density 4–5 times greater than its alkaline counterpart [9]. Electrode reactions in acidic solutions are kinetically faster. Furthermore, acidic electrolyzers display superior resistance to shutdowns and have better load cycling stability [5]. As such, acidic water electrolysis emerges as a more viable method for hydrogen production [10]. Yet, catalysts participating in the anodic oxygen evolution reaction (OER) tend to corrode in these conditions, affecting their stability and activity [11]. Presently, only select precious metal-based catalysts, such as Ir, IrO_2_, and RuO_2_, sustain high activity in acidic water electrolysis [12,13,14]. But while these metals excel in performance, their scarcity, high cost, and gradual dissolution in acidic environments [15,16] limit their broader adoption. This challenge has driven significant research toward non-precious metal catalysts [17], especially abundant transition metals like Fe, Co, Ni, and their derivatives [18,19,20]. These metals are seen as viable alternatives to the precious ones. However, in comparison to their expensive counterparts, they possess inferior catalytic activity and demonstrate high reactivity and instability in acidic settings [21]. Current endeavors aim to boost the performance and endurance of these catalysts in acidic conditions. This article first delves into the OER mechanism of the anode under such environments and later discusses strategies to improve the catalyst’s activity and stability. Table 1 presents an exhaustive list of the acidic OER non-noble metal catalysts reported.

## 2. Oxygen Evolution Reaction Mechanism in Acidic Environments

**Figure 2:**
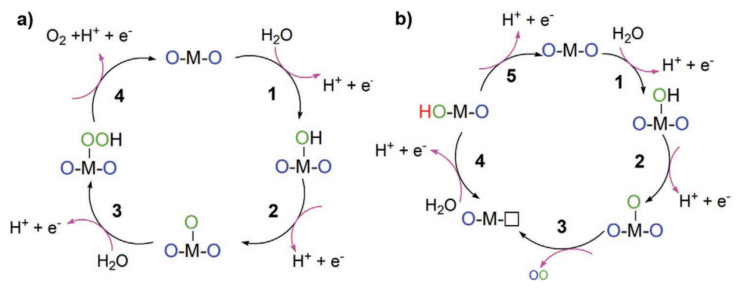
Schematic reaction pathways of acidic OER: (**a**) adsorbate evolution mechanism (AEM) and (**b**) lattice-oxygen oxidation mechanism (LOM). Reproduced with permission. Copyright 2019, American Chemical Society [44].

Anodic water oxidation in an acidic medium encompasses complex electron and proton transfers. During the OER, oxygen molecules arise from several interconnected electron/proton exchanges. The OER’s sensitivity to pH is notable: in acidic settings, it yields four protons (H^+^) and one oxygen molecule. Overcoming kinetic hurdles remains a challenge for OER electrocatalysts. Broadly, the electron transitions during OER in acidic conditions are categorized into four phases [41,42,43]: AEM(a)LOM(b). Refer to Figure 2 [44].

2H_2_O + ^∗^ → OH^∗^ + H_2_O + H^+^ + e^−^ (1)

OH^∗^ + H_2_O → O^∗^ + H_2_O + H^+^ + e^−^ (2)

O^∗^ + H_2_O → OOH^∗^ + H^+^ + e^−^ (3)

OOH^∗^ → O_2_ + ^∗^ +H^+^ + e^−^ (4)

This illustration aptly depicts the OER process. The energy barriers linked to various reaction intermediates stem from the electrocatalyst’s topological and electronic configurations [45].

Broadly, the OER reaction mechanism can be divided into two prevalent pathways: the Adsorption Evolution Mechanism (AEM) and the Lattice Oxygen Mechanism (LOM) [46,47]. AEM stands as the most endorsed model for OER, with its relationship with distinct reaction intermediates being widely recognized [48]. According to the Sabatier principle, the binding strength of intermediates on the catalyst surface primarily determines the overpotential [49]. Although the scaling relations of AEM expedite catalyst selection, challenges in optimizing OER activity persist. With comprehensive experimental and theoretical insights into the reaction mechanism and factors influencing catalyst activity, there is growing evidence of the OER involving lattice oxygen or LOM [50]. LOM circumvents the limitations posed by AEM, suggesting that the active reaction sites extend beyond just the metal core.

### 2.1. Adsorption Evolution Mechanism (AEM)

In the 1990s, various mechanisms, like the oxide route, electrochemical oxide method, and electrochemical metal peroxide strategy, were proposed, primarily based on the Adsorption Evolution Mechanism (AEM) [51]. Within the scope of AEM, the catalyst essentially offers optimal adsorption sites, while the gaseous O product and all involved reaction intermediates arise from the electrolyte [52]. AEM is predominantly understood to encompass four synchronized proton–electron transfer reactions anchored at the metal’s active site [53]. Water molecules initially attach to the metal surface via a one-electron oxidation event, creating an adsorbed *OH at the M site. This *OH subsequently undergoes simultaneous proton and electron release, generating the *O entity. The subsequent O-O bond-formation step allows *O to interact with another water molecule, yielding *OOH. In the end, *OOH partakes in a one-electron transfer event to oxidize, liberating O_2_ and reestablishing the original M active site.

H_2_O + * → *OH + H^+^ + e^−^ ΔG_1_

*OH → *O + H^+^ + e^−^ ΔG_2_

*O + H_2_O → *OOH + H^+^ + e^−^ ΔG_3_

*OOH → * + O_2_ + H^+^ + e^−^ ΔG_4_

**Figure 3:**
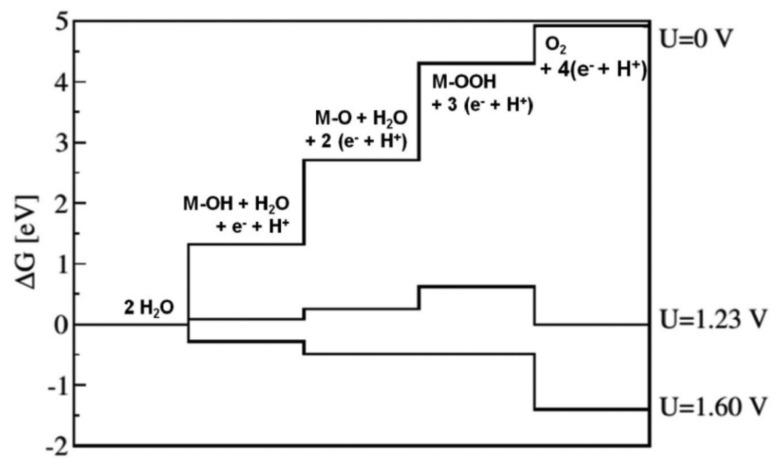
Free energy. Reproduced with permission. Copyright 2007, Elsevier [55].

Every fundamental step possesses a distinct free energy, inherently tied to the binding energy of its corresponding intermediate. The difference in free energy, represented as ∆G, for the step with the most significant value, is identified as the Rate-Determining Step (RDS) and establishes the theoretical overpotential for the entire process (η = (G/e)~1.23V) [54]. From a thermodynamic perspective, an optimal catalyst should display approximately equal reaction free energies across all four stages. As shown in Figure 3.

Under such parameters, the equilibrium potential is set at 1.23 V [56]. Yet, practically, due to the linear scaling relation of intermediate adsorption energies, catalysts do not display this ideal behavior. It has been discerned that both *OH and *OOH symbolize single bonds between the O atom and the catalyst’s surface, predominantly favoring similar binding sites [54]. Consequently, the binding energies of *OH and *OOH scale in a linear manner, characterized by a slope close to 1 and an intercept around 3.2 eV, solely dictated by the interaction of the intermediates with the catalyst’s surface. It is significant to note that the value of ∆G for *OOH can be inferred directly from ∆G for *OH. Their adsorption energies, being independent of the binding energy of *OH, set a base threshold for the OER overpotential. Achieving minimal overpotential is feasible if the energy level of *O is ideally positioned between *OH and *OOH, given the consistent energy disparity between *OH and *OOH’s adsorption energies. Therefore, the OER overpotential is essentially determined by the adsorption energy of *O, indicating that either the second or third primary step functions as the RDS. Hence, the OER overpotential can be articulated through the subsequent equation:

$$\eta =\max[\Delta G_{2},\;\Delta G_{3}]/-1.23\;V\;\;\;\;\;\;\;\;\;\;\;\;\;=\{\max[(\Delta G^{*O}-\Delta G^{*OH}),(\Delta G^{*OOH}-\Delta G^{*O})]/e\}-1.23\;V\;\;\;\;\;=\{\max[\Delta G^{*O}-\Delta G^{*OH}),\;3.2\;\mathrm{ev}-(\Delta G^{*O}-\Delta G^{*OH})]/e\}-1.23\;V$$

**Figure 4:**
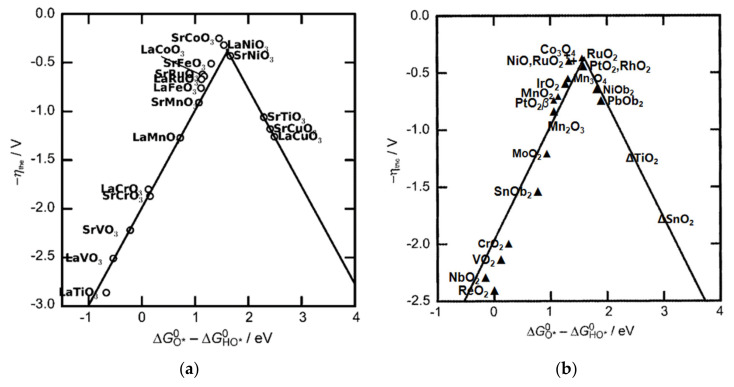
(**a**) Activity trends towards oxygen evolution plotted for perovskites. The negative theoretical overpotential was plotted against the standard free energy of the $\Delta G_{O^{*}}^{0}-\Delta G_{HO^{*}}^{0}$ step. The low coverage regime was considered and the calculated values were used to show the activity of each oxide. The volcano curve was established by using the scaling relation between $G_{HOO^{*}}^{0}-G_{O^{*}}^{0}$ and $G_{O^{*}}^{0}-G_{HO^{*}}^{0}$. (**b**) Activity trends towards oxygen evolution for rutile, anatase, Co_3_O_4_, and Mn_x_O_y_ oxides. The negative values of theoretical overpotential were plotted against the standard free energy of $\Delta G_{HO*}-\Delta G_{O*}$ step. Adapted with permission. Copyright, 2011, John Wiley and sons [54].

Therefore, the difference between the values of ∆G and −∆G serves as the sole descriptor for OER activity, as shown in Figure 4.

Therefore, the difference ∆G_O*_ − ∆G*_OH_* emerges as the sole indicator for OER activity. When plotting η against Δ*G_O_* − Δ*G*_OH_, a universal volcano curve arises, irrespective of the catalyst (as depicted in Figure 4).

**Figure 5:**
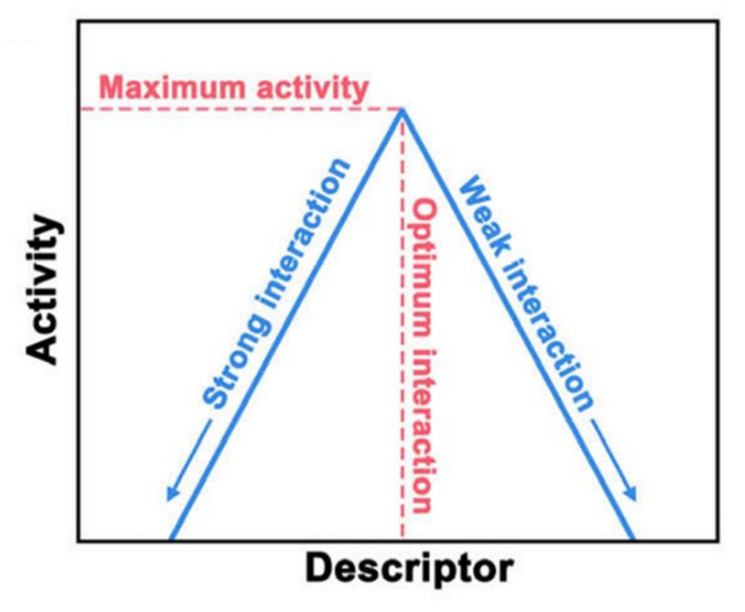
Volcano plot using descriptor to represent the catalytic activity trend. Adapted with permission. Copyright 2021, Elsevier [58].

Numerous scholars are committed to identifying a singular microscopic parameter that encapsulates the OER catalytic activity across diverse electrode materials. This defining parameter is labeled as a descriptor. Such a descriptor might be an inherent characteristic of the catalyst or a byproduct of the interaction between the catalyst and reactants. An effective descriptor invariably reflects the intensity of the relationship between pivotal intermediates in the catalytic process and active sites on the catalyst’s surface region. [57] Utilizing these descriptors, one can draft a volcano curve. Peak activity is attainable at the descriptor’s optimal value (shown in Figure 5), indicative of a balanced interaction.

### 2.2. Lattice Oxygen Mechanism (LOM)

With the growing array of OER catalysts, certain catalytic behaviors have surfaced that are not compatible with the Adsorption Evolution Mechanism (AEM) framework. Contemporary studies have identified materials exhibiting catalytic performances that surpass the boundaries set by the volcano plot, with the activity of certain catalysts being contingent on the pH value measured on the RHE scale [59,60,61]. This discrepancy has prompted theories suggesting that these catalysts might operate through a different reaction mechanism. Consequently, several OER mechanisms have been postulated, with the Lattice Oxygen Mechanism (LOM) receiving considerable attention. LOM entails the lattice oxygen atoms from the catalyst (primarily from the surface region) exiting their structured formation to directly engage in producing dioxygen during OER [52]. The inception of LOM was to sidestep the adsorption scaling relationships inherent in AEM. Recent advancements in molecular-level characterization have indicated that a subset of catalysts, such as Ru/Ir-based catalysts, perovskites possessing high metal–oxygen covalency, and certain Co-based catalysts, demonstrate some degree of lattice oxygen atom participation in OER [62,63]. Due to its novelty, our grasp on the intricate LOM process is still evolving, and its foundational aspects are yet to be fully unraveled.

In the LOM paradigm, the catalyst’s lattice oxygen directly partakes in oxygen evolution and the synthesis of oxygen molecules [64]. The notion of a lattice-involved mechanism was first cited in a 1976 study of PtO catalysts by Damjanovic and Janovic [65]. Later, in 1987, Mehrens and Heitbaum presented corroborating evidence, using isotope labeling to showcase the catalyst’s oxygen contribution to the creation of oxygen molecules [66]. A recent discovery by Mefford et al. posited that the exceptional activity of specific catalysts can be rationalized through the LOM framework, as elucidated by DFT calculations [67].

**Figure 6:**
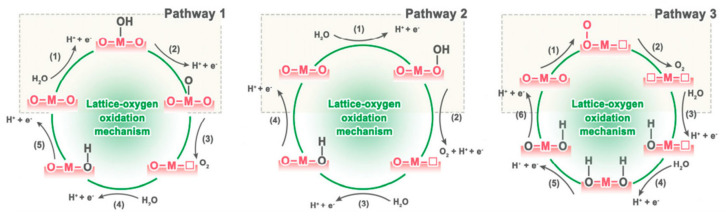
Schematic illustration of three possible LOM schemes. Adapted with permission. Copyright 2021, Elsevier [58].

Historical investigations have put forth three distinct LOM reaction pathways, as illustrated in the diagram in Figure 6 [58]. These pathways primarily diverge in how the lattice oxygen binds with emerging intermediates.

In the initial pathway, the HO* from the water molecule interacts with the metal adsorption point M, leading to oxidation to form O*. This process mirrors the initial steps in AEM. Subsequently, O* reacts directly with the lattice oxygen, producing dioxygen OO*, which releases the O_2_ molecule and creates an oxygen vacancy on the catalyst’s surface. The adsorption of HO* can refill this vacancy, thereby maintaining the catalyst’s structural integrity. In the alternative pathway, the OH* intermediate favors the lattice oxygen site over the metal site for initial adsorption. This suggests that HO* binds directly with the lattice oxygen, forming HOO*. This HOO* then dissociates to yield O_2_ and H^+^. The lattice oxygen partially contributes to the resulting O_2_ molecule. In a distinct third pathway, two lattice oxygen atoms may join under OER conditions, generating twin oxygen vacancies on the catalyst’s surface.

Differential Electrochemical Mass Spectrometry (DEMS), supplemented by ^18^O isotope labeling experiments, offers insights into the LOM mechanism within acidic environments.

### 2.3. Difference of Oxygen Evolution Reaction Mechanism under Acidic and Alkaline Conditions

Due to the different substances participating in the reaction under different media conditions, the mechanisms of OER are different. It is generally believed that the reaction process is as follows: in the base electrolyte, OH- is first adsorbed on the surface of the electrode catalyst and loses an electron to form M-OH*. Then, with the participation of OH-, M-OH* loses an electron and is oxidized to M-O, respectively. * and M-OOH*, M-OOH* finally combines with OH- to form oxygen and desorb. The specific OER reaction mechanism is shown below [68,69,70]:

OH^−^ + ^∗^ → OH^∗^ + e^−^ (5)

OH^∗^ + OH^−^ → O^∗^ + H_2_O + e^−^ (6)

O^∗^ + OH^−^ → OOH^∗^ + e^−^ (7)

OOH^∗^ + OH^−^ → O_2_ + ^∗^ + H_2_O + e^−^ (8)

Comparing the reaction Gibbs free energies of the acidic and alkaline reaction mechanisms, it can be seen that the only difference lies in the electrode potential. In the case of the acidic mechanism, the electrode potential is related to the SHE, and in the case of the alkaline mechanism, it is related to the RHE. Hence, the reaction Gibbs free energies can be calculated with almost the same equations even though the reaction mechanisms for the acidic environment and for the alkaline environment are different.

## 3. Strategies for Catalyst Activity Enhancement

The inherent slow kinetics result in the reduced conversion efficiency of acidic OER catalysts. Augmenting the performance of acidic OER is pivotal for the future of PEMWE technologies. At a high level, the key approaches to refine OER catalysts involve amplifying the count of active sites and bolstering the activity per site. Ideally, these methods can be pursued in tandem, yielding a marked surge in performance. Innovative methods to elevate the effectiveness of acidic OER catalysts have been explored, encompassing compositional refinements, morphological innovations, atomic infusions, defect modifications, induced strains, and anionic adjustments. The final catalyst morphology, surface composition, and electronic nuances profoundly influence these strategies, making them essential for sculpting the optimal catalyst. More often than not, these elements interplay rather than function independently, synergistically affecting the catalyst’s performance.

### 3.1. Compositional Refinement

The catalyst’s composition, particularly its surface composition, critically influences its activity [71]. Through synergistic interactions, enhanced coordination settings, and the electronic configurations of multi-metallic entities, the characteristics of mono-metallic materials can be sophisticatedly tailored [72]. As such, strategically fine-tuning the catalyst’s composition emerges as a pivotal method for engineering potent acidic OER catalysts [73]. Two principal techniques can modulate this composition. Firstly, ex situ material synthesis methods, which encompass atomic doping and alloying, offer means for composition adaptation [74,75]. For instance, integrating heteroatoms into IrO_2_ or crafting Ir-centric alloys stand as exemplars [76,77].

**Figure 7:**
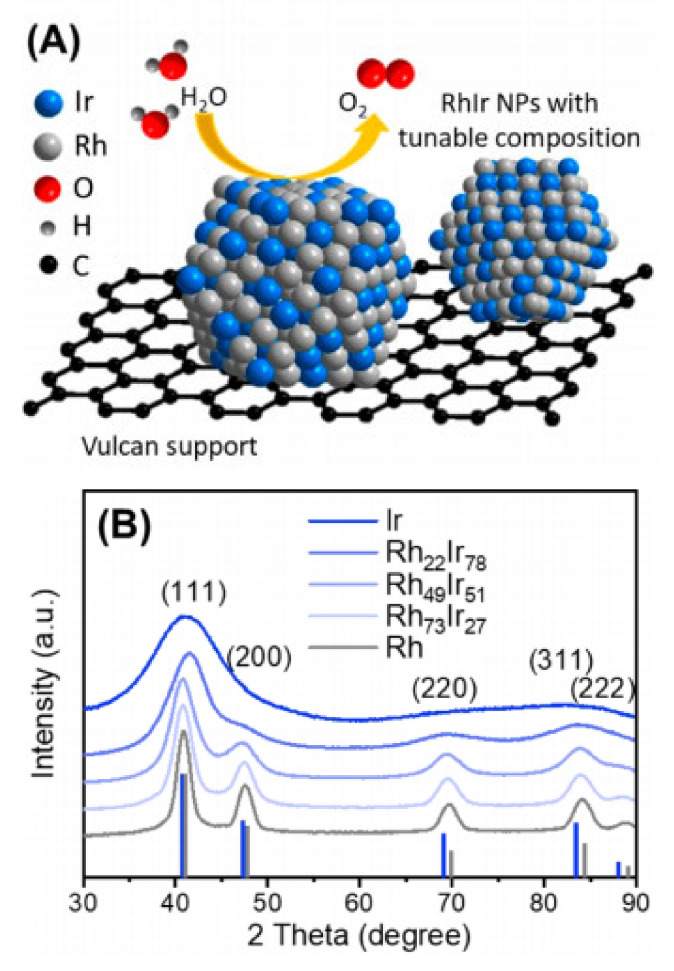
(**A**) Illustrative depiction of Rh_x_Ir_(100−x)_ nanoparticles anchored on Vulcan XC-72R carbon, purposed for OER in acidic conditions. (**B**) PXRD patterns of Ir nanoparticles, Rh nanoparticles, and Rh_x_Ir_(100−x)_ nanoparticles with varying compositions. Established PXRD reflection positions for Ir and Rh are also presented for comparison. Adapted with permission. Copyright 2019, American Chemical Society [74].

Guo and colleagues unveiled a method using microwave-assistance to synthesize Rh_x_Ir_(100−x)_ alloy nanoparticles spanning an extensive compositional spectrum (x = 22–73) [74]. Although pristine Rh showcased a comparatively subdued OER activity relative to pure Ir, the electron strain dynamics instigated by alloying were anticipated to tweak the adsorbate adhesion on the pure Ir sites across the Rh-Ir alloy surface. Such combined alloying influences are primed to refine the active Ir locales for OER, leading to amplified OER action. These particles exhibited exceptional potency and longevity in acidic solutions (refer to Figure 7). Among the array of Rh_x_Ir_(100−x)_ nanoparticles, the Rh_22_Ir_78_ variant displayed an apex mass activity of 1.17 A mg^−1^ Ir and a commensurate TOF rate of 5.10 s^−1^—metrics that rank among the elite for acidic OER catalysts. After enduring 2000 OER cycles, only a marginal shift in the polarization curve manifested, testifying to the robustness of the Rh-Ir compound. Further, Density Functional Theory (DFT) analyses inferred that amalgamating a minimal Rh quantity (22 atomic%) with Ir yielded the minimal binding energy disparity between the O and OOH intermediaries, expediting the OER’s pivotal step and bolstering its efficiency. This investigation not only charts a voyage into the judicious architecture of the alloy nanoparticle framework but also accentuates the imperative of meticulously adjusting catalyst composition to magnify electrocatalytic OER outcomes. Powder X-ray diffraction (PXRD) analyses corroborated that Rh-Ir nanoparticles adopt a face-centered cubic (FCC) configuration, mirroring the structural attributes of unadulterated Ir and Rh (See Figure 7).

**Figure 8:**
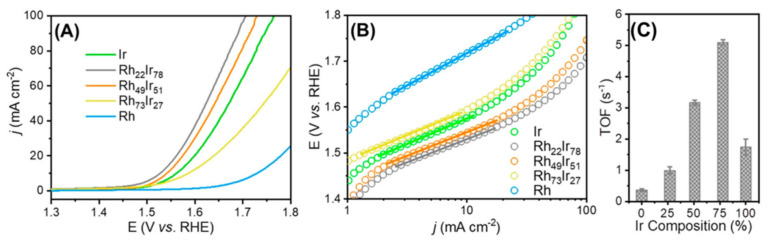
(**A**) IR-adjusted OER polarization curves of Ir/VXC, Rh/VXC, and Rh_x_Ir_(100−x)_/VXC across varying compositions, gauged in a 0.5 M H_2_SO_4_ aqueous medium. (**B**) Tafel plots representing the electrocatalysts. Both experimental and theoretical research indicate that the incorporation of 22% Rh into Ir culminates in a reduced binding energy variance between the O and OOH intermediaries. (**C**) Turnover frequencies normalized by the ECSA for different catalysts at 1.53 V vs. RHE. This translates to a remarkable enhancement in OER efficacy: a decline of 48 mV in overpotential to achieve a current density of 10 mA cm^−2^ and a tripling of mass activity in comparison to similar Ir NP catalysts. Post-2000 cycles, there was an absence of any notable dip in OER vigor, underscoring the robust stability of the carbon-backed Rh-Ir alloy nanoparticles in acidic settings. Adapted with permission. Copyright 2019, American Chemical Society [74].

All electrochemical evaluations took place in a 0.5 M H_2_SO_4_ aqueous medium post-catalyst activation, utilizing a scan velocity of 10 mV s^−1^. IR-adjusted polarization diagrams revealed the hierarchy of OER catalytic efficacy across diverse composite catalysts as follows: Rh_22_Ir_78_/VXC outperformed Rh_49_Ir_51_/VXC, which in turn was superior to Ir/VXC, followed by Rh_73_Ir_27_/VXC, with Rh/VXC trailing last (Refer to Figure 8A). This intimates that, in juxtaposition with pristine Ir nanoparticles, those with a higher Ir concentration showcased a marked augmentation in OER efficacy. In addition, Rh-dominant Rh-Ir electrocatalysts surpassed the performance of unalloyed Rh nanoparticles. Significantly, of all the tested blends, the premier performer, Rh_22_Ir_78_/VXC, manifested the most diminutive Tafel gradient (101 mV dec^−1^), underscoring an elevated charge transfer quotient and brisker kinetic dynamics (See Figure 8B and Table 2). Furthermore, it required an overpotential of only 292 mV to achieve a current density of 10 mA cm^−2^, which is 48 mV lower than the 340 mV overpotential of Ir/VXC (Table 2). The intrinsic activity of Rh_x_Ir_(100−x)_ NPs was then evaluated by calculating the ECSA-normalized TOFs. The resulting ECSA-normalized TOFs displayed the same trend as the mass activities (See Figure 8C and Table 3). These results confirm that there is a direct influence of surface Ir concentration on OER activity.

### 3.2. Morphological Engineering

The morphology of a catalyst significantly influences its activity. Predominantly, OER transpires on the exterior and adjacent subsurface sectors of the catalyst [78]. Hence, materials boasting expansive surface areas can unveil a greater count of active locales, thereby augmenting catalytic prowess [79]. Techniques like crafting nano-porous svelte structures and 3D designs aim to amplify surface area and proliferate the tally of discernible active sites.

#### 3.2.1. Nano-Porous Constructs

**Figure 9:**
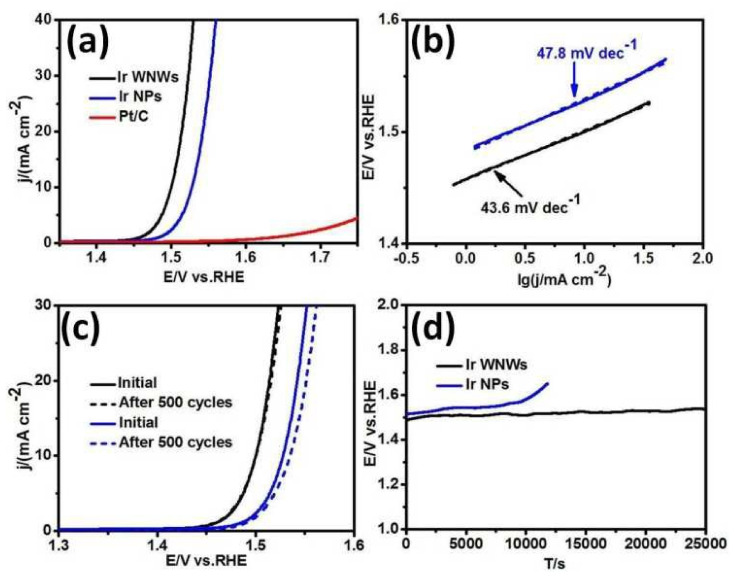
(**a**) Depicting the OER polarization curves and (**b**) associated Tafel plots for Ir WNWs, Ir NPs, and Pt/C in a 0.5 M HClO_4_ acidic milieu. (**c**) The LSV curves are provided for both Ir WNWs (illustrated in black) and Ir NPs (in blue) in the 0.5 M HClO_4_ medium. The solid line portrays the inaugural cycle, while the dotted line illustrates the 500th cycle. (**d**) A chronopotentiometry analysis juxtaposes the performance of Ir WNWs against Ir NPs in a 0.5 M HClO_4_ medium, maintaining a current density of 5 mA cm^−2^. Reproduced with permission. Copyright 2009, Royal Society of Chemistry [81].

Nano-porous entities reveal an extensive catalyst-to-electrolyte interface, thus offering a more copious assembly of active sites to steer the reaction [80]. Fu and team devised an innovative undulating Ir nanowire with a breadth of 1.7 nm via a wet chemical technique [81]. This singular ultra-slender rippling nanoconstruct, possessing a pronounced aspect ratio coupled with an ample specific surface expanse, bolstered OER efficacy significantly. In a 0.1 M HClO_4_ medium, the overpotential to attain 10 mA cm^−2^ stood at a mere 270 mV, noticeably superior to Ir nanoparticles. Assessing the OER potential of IrWNWs, IrNPs, and Pt/C catalysts entailed electrochemical trials in both 0.5 M and 0.1 M HClO_4_ mediums. As depicted in Figure 9a, amongst the trio of assessed catalysts, Ir WNWs reigned supreme, flaunting an optimal catalytic activity with a paltry overpotential of 270 mV to reach a current density of 10 mA cm^−2^ in 0.5 M HClO_4_, clearly outstripping Ir NPs (298 mV). Additionally, the Tafel gradient for Ir WNWs was pegged at 43.6 mV dec^−1^, outshining Ir NPs at 47.8 mV dec^−1^, as illustrated in Figure 9b. Predictably, Pt/C registered a sizably escalated overpotential under identical parameters, signaling its markedly diminished OER capacity. Figure 9c lays out the cyclical resilience of the forged Ir catalysts. After a 500-cycle run in a 0.5 M HClO_4_ milieu, Ir WNWs’ efficacy remained largely unaltered, while the overpotential for Ir NPs escalated by 8 mV at the same current density. An endurance analysis of the Ir WNWs catalyst, conducted via a chronoamperometric technique, is showcased in Figure 9d. Maintaining a steady current density of 5 mA cm^−2^, Ir WNWs manifested negligible wear across an unbroken electrolysis span of 25,000 s. In contrast, the resilience of Ir NPs was distinctly lackluster.

#### 3.2.2. Three-Dimensional Architectures

Recent advances in catalysis have brought forth a slew of three-dimensional (3D) catalyst configurations. This includes the likes of 3D Ir superstructures [82], the core-shell design of IrO_2_@RuO_2_ [83], the intricately designed 3D nano-porous Ir_25_Os_75_ alloy [84], and the novel Cu-doped RuO_2_ hollow porous polyhedrons [85,86]. Under acidic ambiances, these 3D setups have shown promise as potent OER catalysts. The underpinning reason is that these 3D designs afford a plethora of active sites, thus boosting the overall catalytic activity.

Let us consider the 3D Ir superstructure for deeper insights. This meticulously crafted structure is a product of a wet chemical procedure. It comprises exceptionally thin Ir nanosheets acting as foundational sub-units, cumulatively giving rise to a 3D ensemble. The merits of this configuration are multifaceted: it avails an expansive and accessible 3D active site portfolio, offers an enlarged surface area, has an apt interlayer spacing, and its superstructure architecture is decidedly favorable for bolstering electrochemical energy transitions. Its performance under acidic conditions is commendable, showcasing an impressive OER proficiency with a modest onset overpotential and a diminutive Tafel gradient of just 40.8 mV dec^−1^.

**Figure 10:**
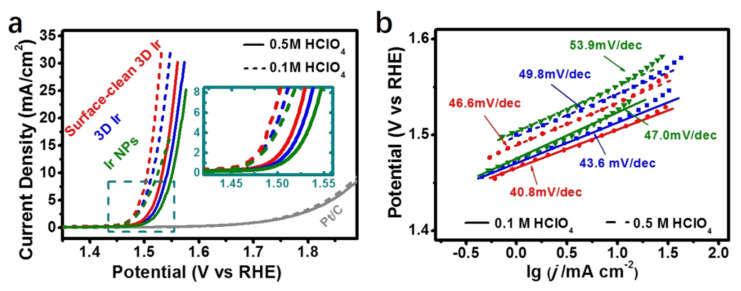
Electrochemical performance of 3D Ir superstructure, surface-clean 3D Ir superstructure, Ir NPs, and Pt/C (JM 20%) catalysts in acidic condition. (**a**) The polarization curves and (**b**) Tafel plots of 3D Ir superstructures, surface-clean 3D Ir superstructures, Ir NPs, and Pt/C (JM 20%) catalysts in 0.1 and 0.5 M HClO_4_ solutions with 95% iR-compensation at the scan rate of 1 mV s^−1^. Adapted with permission. Copyright 2016, American Chemical Society [82].

To meticulously gauge the OER potential of this 3D Ir superstructure in acidic milieus, rigorous tests were orchestrated in both 0.1 M and 0.5 M HClO_4_ mediums. Observations revealed that both the surface-pristine 3D Ir and its counterpart, the surface-cleaned 3D Ir superstructure, had onset potentials that were nearly indistinguishable (as per Figure 10a). Yet, the latter showcased heightened catalytic zeal at amplified overpotentials—a feat credited to its more expansive Electrochemical Active Surface Area (ECAS). When probed in 0.1 M HClO_4_, this surface-purified 3D Ir superstructure necessitated an overpotential of a mere 0.27 mV to touch 10 mA cm^−2^. This performance clearly surpassed both its uncleaned twin and the Ir NPs. Pt, on the other hand, consistently displayed subpar OER efficacy in these acidic setups. Adding another feather to its cap, the surface-cleaned 3D Ir superstructure recorded an exceptionally low Tafel slope, just 40.8 mV dec^−1^ in acidic medium (as seen in Figure 10b). This performance is noticeably superior when juxtaposed with the likes of IrO_2_ and RuO_2_.

### 3.3. Electronic Structure

#### 3.3.1. Defect Engineering

**Figure 11:**
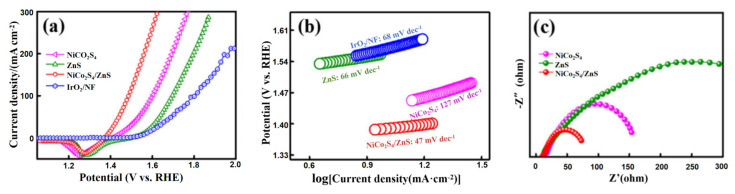
(**a**) OER polarization curves, (**b**) Tafel slopes, and (**c**) Nyquist plots measured at 0.4 V for various catalysts. Adapted with permission. Copyright, 2021, John Wiley and sons [88].

Defects play a pivotal role in determining the physicochemical attributes of materials and have thus become indispensable in the realm of catalysis [70,87,88,89]. Defect engineering, in particular, is crucial for designing high-performance carbon nanomaterials tailored for diverse energy conversion and storage applications [90]. Properly introduced defects can optimize the surface chemistry of active sites, modulate the adsorption of reaction intermediates, and consequently amplify catalytic activity [91]. This realm of engineering spans adjustments in electronic structures at molecular or atomic scales, such as lattice distortion [92], intentional vacancies, and uncoordinated sites [85,93]. Defects can be introduced through various experimental techniques, including heteroatom doping [94], foreign element substitution [95], element extraction [96], atomic etching [97], and heterointerface generation [98]. Numerous efficient catalysts have emerged from these techniques. For instance, Sunjing presented a design where Zn-deficient ZnS nanospheres adorned NiCo_2_S_4_ nanosheet surfaces, forming a NiCo_2_S_4_/ZnS heterostructure [88]. This unique integration of a heterointerface with metal defects allows for tailoring the local electronic structure, enhancing the catalyst’s efficacy. The attachment of these ZnS nanospheres countered the volume expansion of NiCo_2_S_4_ nanosheets during operation, fortifying the composite’s structural integrity. The synthesized NiCo_2_S_4_/ZnS heterostructure showcased outstanding OER performance, necessitating only a 140 mV overpotential with a Tafel slope of 47 mV dec^−1^, ranking it among the elite metal sulfides. Density functional theory (DFT) insights revealed that the intrinsic interface potential combined with Zn defects expedited electron movement, reshaping the electronic landscape and bolstering catalytic efficiency. Linear sweep voltammetry (LSV) analyses highlighted that NiCo_2_S_4_/ZnS delivered superior OER activity, achieving a 140 mV overpotential at 10 mA cm^−2^, which is notably lower than that of NiCo_2_S_4_ (220 mV), ZnS (320 mV), and even commercial IrO_2_ (340 mV) as illustrated in Figure 11a. Furthermore, NiCo_2_S_4_/ZnS’s Tafel slope stood at 47 mV dec^−1^, outperforming NiCo_2_S_4_ (127 mV dec^−1^), ZnS (66 mV dec^−1^), and IrO_2_ (68 mV dec^−1^), suggesting NiCo_2_S_4_/ZnS boasts swift OER kinetics as illustrated in Figure 11b. Electrochemical impedance spectroscopy (EIS) evaluated charge transfer attributes. Figure 11c displayed that the Rct value for the NiCo_2_S_4_/ZnS catalyst was around 75 Ω, underscoring its rapid kinetic response.

#### 3.3.2. Strain Engineering

**Figure 12:**
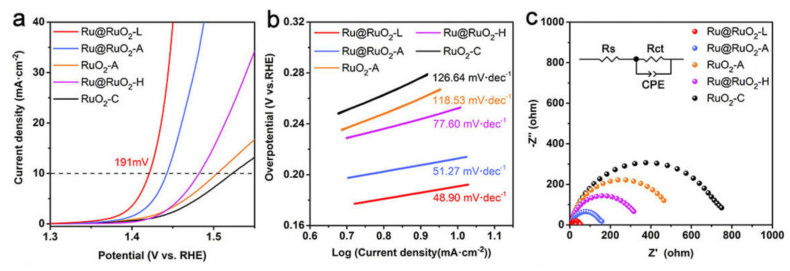
Electrochemical performance of RuO_2_, RuRh NS, and RuRh@(RuRh)O_2_ NS in O_2_-saturated 0.1 M HClO_4_. LSV curves of the (**a**) RuRh NS and (**b**) RuRh@(RuRh)O_2_ NS in 0.1 M HClO_4_ at 1600 rpm with scan rate 10 mV s^−1^ at the first few cycles. (**c**) LSV curves of the first cycle. Adapted with permission. Copyright 2020, Elsevier [103].

Lattice strain arises when atoms or particles on a material’s surface or within certain areas deviate from their expected atomic configurations or distances in the bulk material [99]. Such deviations, be they lattice vacancies, distortions, or mismatches, can influence the electronic structure [100]. Strain engineering is a deliberate strategy to introduce lattice strain to modify the electronic makeup of a catalyst, thereby optimizing the adsorption of reaction intermediates [101,102]. In the realm of acidic OER catalysts, two prominent applications of strain engineering emerge: the crafting of core-shell architectures and the development of grain boundaries. A case in point is the Ru@RuO_2_-L core-shell nanoparticles, wherein the RuO_2_ shell layer undergoes tensile strain, modulating the charge density of Ru(IV) to bolster its acidic reactivity [103]. The Ru@RuO_2_-L catalyst was adeptly crafted through a single-step laser irradiation technique. High-resolution spectroscopy detected a 6% tensile strain in the Ru-O bonds, which, in turn, diminished the charge density of Ru(IV). Consequently, the emerging Rux^+^ active sites (where 4 < x < 5) substantially hastened the oxygen evolution reaction (OER) in acidic settings. The performance of Ru@RuO_2_-L in acidic OER was evaluated in a 0.5 mol L^−1^ H_2_SO_4_ solution employing a standard three-electrode electrochemical setup. The linear sweep voltammetry (LSV) profile for OER, as showcased in Figure 12a, sees an uptick in the current, signaling the commencement of OER. Notably, Ru@RuO_2_-L demonstrated superior OER proficiency, achieving a minimal overpotential of 191 mV, corresponding to a 10 mA cm^−2^ current density. This is markedly less than the commercial RuO_2_ catalyst’s overpotential of 293 mV. Moreover, Ru@RuO_2_-L also boasts the most minimal Tafel slope at 48.9 mV dec^−1^, underscoring a pronounced enhancement in OER kinetics in acidic conditions (refer to Figure 12b). The EIS plot in Figure 12c reveals Ru@RuO_2_-L with the tiniest semicircle radius, suggesting optimal charge transfer speeds and further accentuating its remarkable OER kinetics.

## 4. Strategies to Enhance Catalyst Stability

Beyond mere activity, stability emerges as a vital criterion in gauging the efficacy of an OER catalyst. Given the abrasive environment, especially in acidic electrolytes, ensuring the prolonged durability of OER catalysts becomes crucial [104,105]. Operating under these stringent acidic conditions, catalysts are expected to retain their robustness throughout extended cycles of reactions. Crafting a catalyst that marries both exceptional stability and activity is often a daunting endeavor [106,107,108]. A prevalent observation is the apparent trade-off between OER activity and stability [5]. Danilovic and colleagues’ investigation into the interplay between activity and stability across an array of metal oxides (Os, Ru, Ir, Pt, Au) during OER serves as a testament to this [109]. To fortify catalyst stability, several strategies have been championed. These encompass tailoring the alloy structure to recalibrate the reaction pathway for enhanced stability [110], leveraging support stabilization, introducing protective layers, fine-tuning morphology and phase, and tweaking electronic/atomic/nano-configurations.

### 4.1. Refining Structure and Composition

**Figure 13:**
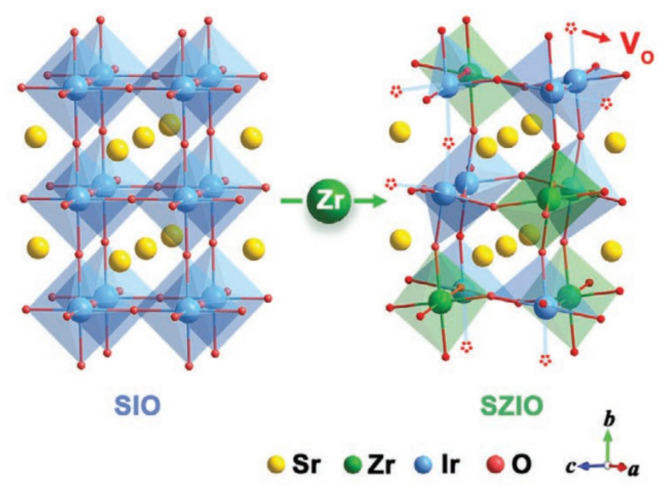
Schematic illustration of the structures of SIO and SZIO. Adapted with permission. Copyright, 2020, John Wiley and sons [111].

One prevalent method is the amalgamation of two high-performing catalysts [109]. In a noteworthy study, Liang’s group introduced the catalytically passive SrZrO_3_ (SZO) to SrIrO_3_ (SIO), culminating in a novel perovskite-style solid–solution electrocatalyst [111]. Among the variants synthesized, the perovskite solid–solution bearing a Zr:Ir atomic composition of 1:2 showcased exemplary catalytic prowess and was christened as (SZIO) (as illustrated in Figure 13)

**Figure 14:**
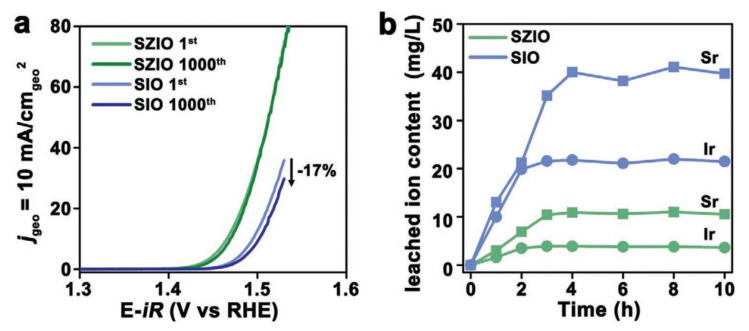
(**a**) LSV curves obtained with SZIO and SIO before and after 1000 CV cycles. (**b**) Contents of leached metals in the electrolyte in the presence of SZIO and SIO during 10 h long electrocatalysis. Copyright, 2020, John Wiley and sons [111].

SZIO exhibited noteworthy stability during acidic OER. As depicted in Figure 14a, following 1000 cyclic voltammetry (CV) iterations, the current density of SIO diminished by 17%, whereas the linear sweep voltammetry (LSV) trajectory for SZIO remained virtually consistent. With sustained catalytic activity at a current density of 10 mA cm^−2^ spanning beyond 30 h, SZIO outperformed SIO in terms of resilience (as illustrated in Figure 14b). This underscores the potential of integrating SZO with SIO to bolster OER stability in acidic environments.

Researchers have posited that by amalgamating IrO_x_ into RuO_x_, which inherently possesses heightened catalytic activity but a swifter corrosion rate, the resultant catalyst could offer improved stability [109,112,113]. Subsequent experiments have validated this theory, revealing that RuxIr_1−x_O_2_ markedly outshines RuO_x_ in terms of durability [114]. In the dynamics of RuxIr_1−x_O_2_, surface Ru ions have a propensity to dissolve, consequently exposing the surface iridium oxide (IrO_x_), which acts as a robust protective layer and remains highly active in acidic environments [77]. Such findings underscore the efficacy of devising core-shell structures with a corrosion-resistant outer layer in bolstering electrocatalytic resilience [115].

**Figure 15:**
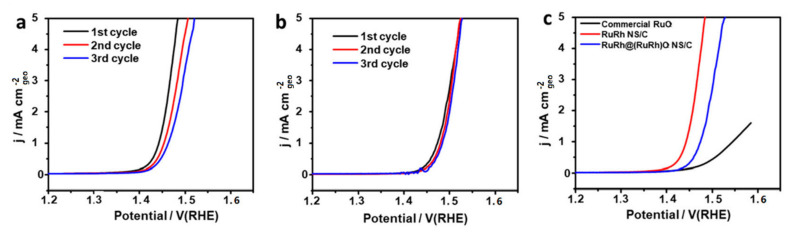
Electrochemical performance of RuO_2_, RuRh NS, and RuRh@(RuRh)O_2_ NS in O_2_-saturated 0.1 M HClO_4_. LSV curves of the (**a**) RuRh NS and (**b**) RuRh@(RuRh)O_2_ NS in 0.1 M HClO_4_ at 1600 rpm with scan rate 10 mV s^−1^ at the first few cycles. (**c**) LSV curves of the first cycle. Reproduced with permission. Copyright 2012, Royal Society of Chemistry [116].

In a related development, Wang et al. [116] unveiled a RuRh@RuRhO_2_ core-shell nanoplate positioned as a steadfast OER electrocatalyst. As depicted in Figure 15a, the RuRh NS exhibited mediocre OER stability. However, in stark contrast, RuRh@RuRhO_2_ NS manifested considerably elevated stability relative to its RuRh NS counterpart, a contrast sharply highlighted in Figure 15b. The comprehensive linear sweep voltammetry (LSV) assessments are showcased in Figure 15c.

**Figure 16:**
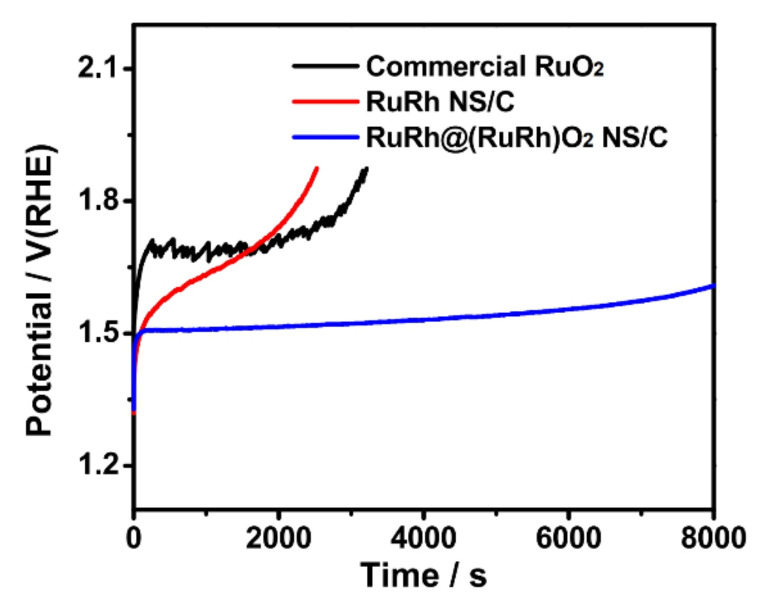
Chronopotentiometry tests of the catalysts in 0.1 M HClO_4_ at 5 mA cm^−2^. Reproduced with permission. Copyright 2012, Royal Society of Chemistry [116].

Chronoamperometry was employed to further probe the stability of RuRh@RuRhO_2_ NS, maintaining a consistent current of 5 mA cm^−2^ (as shown in Figure 16). The analysis accentuated the remarkable stability of RuRh@RuRhO_2_ NS compared to RuRh NS. Utilizing inductively coupled plasma mass spectrometry (ICP-MS), researchers gauged the dissolution rate of Ru in the electrolyte throughout the OER process. Intriguingly, the Ru dissolution rate from RuRh NS stood at 4.0 ng cm^−2^s^−1^, a rate that dwarfs the 0.06 ng cm^−2^s^−1^ dissolution rate of RuRh@RuRhO_2_ NS by a staggering factor of 66.7. Such findings underscore the protective prowess of the RuO_2_/RhO_2_ shell, shielding the metallic RuRh NS core against dissolution during the OER activity. This aligns seamlessly with the insights gleaned from RRDE studies. As a result, RuRh@RuRhO_2_ NS emerges as a distinctly superior OER catalyst in terms of stability relative to RuRh NS.

### 4.2. Curbing Lattice Oxygen Oxidation

To prevent catalyst deactivation, it is imperative to avert the dissociation of lattice oxygen paired with metal sites. Such oxidation results in the creation of oxygen vacancies, which can further spur the disproportionate oxidation of metal atoms. Therefore, fostering a robust oxygen coordination environment becomes instrumental in suppressing the extraction of lattice oxygen, thereby upholding catalyst performance [117].

#### 4.2.1. Metal–Oxygen Orbital Interplay

It is posited that heightened TM d-O p orbital hybridization or covalency within oxides can catalyze lattice oxygen oxidation. This dynamic involves the genesis of oxygen vacancies (V0), amplifying OER activity. Yet, pushing covalency to extremes might induce surface amorphization or undesired structural shifts [118]. Such transitions manifest when the OH- (base)/H_2_O (acid) adsorption pace or the lattice oxygen migration speed cannot counterbalance the surface Vo generation rate, culminating in cation loss [119]. As TM d-O p covalency intensifies, so does the metal dissolution rate, potentially ushering in surface modifications or complete structural disintegration. Numerous studies have flagged excessive metal–oxygen covalency and the ensuing A-site cation dissolution as primary culprits undermining OER stability in acidic milieus [120,121,122].

Yang and his team’s research underscores the instability stemming from excessive metal–oxygen covalency. Their investigations spotlighted how the pyrochlore-type Y_2_Ru_2_O_7−δ_ electrocatalyst outstripped the stability of pure RuO_2_. This enhancement was ascribed to the diminished overlap between Ru 4d and O p orbitals’ band centers. The implication is that yttrium ions (Y3^+^) fortify RuO_x_, furnishing a more resilient structure with optimally poised energy levels under acidic conditions [123].

**Figure 17:**
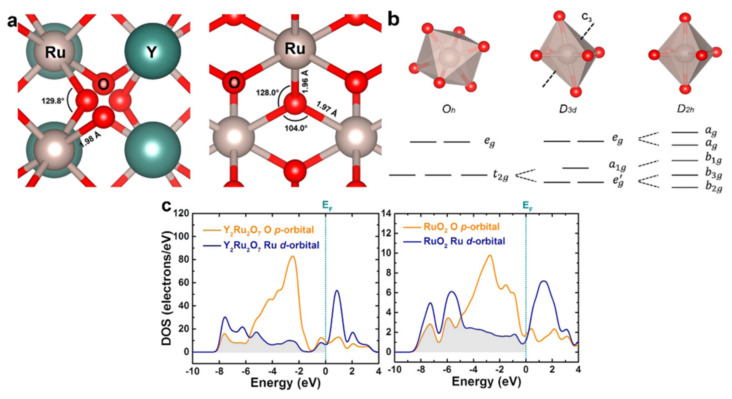
DFT calculation of energy states of Y_2_Ru_2_O_7_ and RuO_2_. (**a**) Local structures showing the Ru−O bond lengths of Y_2_Ru_2_O_7_ and RuO_2_, respectively. (**b**) Illustrations of distorted RuO_6_ structures and 4d orbital splits in octahedral (Oh), trigonal antiprism (D3d), and compressed octahedral (D2h) ligand fields. (**c**) Calculated PDOS plots of Ru 4d and O 2p orbitals for Y_2_Ru_2_O_7_ and RuO_2_. Shaded area shows the overlapped bands between Ru 4d and O 2p orbitals. The Fermi level is set to zero. Copyright 2020, American Chemical Society [123]. High-Performance Pyrochlore-Type Yttrium Ruthenate Electrocatalyst for Oxygen Evolution Reaction in Acidic Media by Jaemin Kim, Pei-Chieh Shih, Kai-Chieh Tsao, Yung-Tin Pan, Xi Yin, Cheng-Jun Sun, and Hong Yang is licensed under CC BY 4.0.

Exemplified in Figure 17, panels (a, b) show that despite the Ru-O bond lengths being analogous across both catalysts, their distinct local positioning alters the Ru 4d orbital energy level in Y_2_Ru_2_O_7−δ_ from that in RuO_2_. Probing deeper into the ramifications of the electronic band structure on OER behavior, DFT computations, specifically the projected density of states (PDOS) of Ru and O atoms, were employed. Panel (c) elucidates that structurally, the band center energy gap separating O 2p and Ru 4d orbitals in Y_2_Ru_2_O_7_ distances itself further from the Fermi level. This positioning likely augments catalytic stability, paralleling the O 2p band center’s positioning.

#### 4.2.2. Doping to Bolster Stability

Doping, the introduction of impurities into a semiconductor, can significantly affect the properties of materials, including OER catalysts. One of the merits of doping in the context of catalysts is its ability to elevate the energy barrier, restricting the formation of oxygen vacancies. This, in turn, fortifies the catalyst’s stability [124].

Take, for instance, the case of Ti-doped SrIrO_3_ in acidic OER processes. Such doping diminishes metal leaching from the oxides, culminating in a catalyst with enhanced durability [125]. Similarly, the application of single-atom Ag in modifying IrO_x_ reinforces the Ir-O lattice oxygen bond. This tightening of the bond reduces the likelihood of lattice oxygen being siphoned away even when the overpotential is low, ensuring improved OER stability [126]. Another prime example is W and Er co-doped RuO_2_ (W_0.2_Er_0.1_Ru_0.7_O_2−δ_), which stands as a formidable catalyst for acidic OER [124]. It demands a mere overpotential of 168 mV at 10 mA cm^−2^ and has an impressive stability record spanning 500 h. The integral role of W and Er doping here is evident—they significantly inhibit the formation of soluble Rux > 4 and diminish the adsorption energy of oxygen intermediates, constraining the extraction of lattice oxygen. These measures collectively amplify stability.

### 4.3. Electronic Coupling: Key to Longevity

For any catalyst, the choice of substrate plays a paramount role, especially in acidic OER catalysts aiming for extended operations. A recurring challenge during the OER process is that some active sites on the substrate’s surface may experience detachment, undermining performance [127]. Thus, a foundational trait of ideal substrates is their proficiency in exhibiting potent electronic coupling with the loaded precious metals. Such coupling averts deactivation which might arise due to the active materials shedding, thereby amplifying stability.

**Figure 18:**
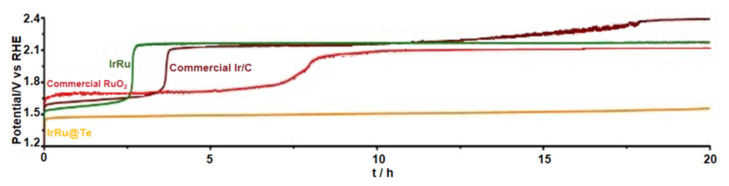
Chronopotentiometric curves of IrRu@ Te and other control catalysts recorded at a constant current density of 10 mA cm^−2^ in 0.5 M H_2_SO_4_. Adapted with permission. Copyright 2020, American Chemical Society [128].

Highlighting this, Xu and team [128] unveiled an IrRu intermetallic compound nanocluster, intricately loaded onto conductive, acid-resistant, amorphous tellurium nanoparticles, christened IrRu@Te. This compound realized through the hydrothermal method, capitalized on the expansive electrocatalytic surface area of the IrRu cluster. Paired with the formidable electronic coupling between IrRu and the Te substrate, the resultant IrRu@Te catalyst exhibited exemplary catalytic performance for OER in a robust acidic electrolyte (specifically, 0.5 M H_2_SO_4_), as illustrated in Figure 18. To put it in numbers, it demanded overpotentials of just 220 and 303 mV to kickstart OER at 10 and 100 mA cm^−2^, respectively. Moreover, it upheld electrolysis at a consistent 10 mA cm^−2^ current density for a sustained 20 h. A clear testament to its dual prowess: high activity paired with robust stability.

**Figure 19:**
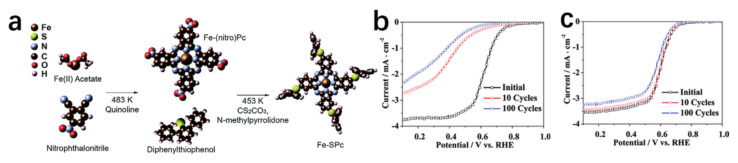
(**a**) Schematic diagram of Fe–SPc synthesized from Fe–Pc. Reproduced with permission. Copyright 2014, Royal Society of Chemistry. (**b**) The accelerated durability test of Fe–Pc. (**c**) The LSV curves of Fe–SPc before and after 10 and 100 cycles. (**b**,**c**) Reproduced with permission. Copyright 2010, American Chemical Society [129].

To enhance the electronic coupling between the carbon matrix and the active sites, thereby preventing Fe dementalization, Li et al. [129] substituted the base with diphenyl phenyl sulfide. This modification of iron phthalocyanine (FePc) resulted in the creation of a notably stable oxygen reduction catalyst, termed Fe-SPc, depicted in Figure 19a. Utilizing the rotating disk electrode (RDE) technique, they assessed the performances of both Fe-PC and Fe-SPC in an oxygen-saturated 0.1 M HClO_4_ solution. Post 10 and 100 scan cycles, the current density of Fe-SPc at 0.5 V versus RHE was 4.6 and 7.4 times greater than that of Fe-Pc, in that order. Despite Fe-SPc’s marginally reduced catalytic activity in contrast to Fe-Pc, it showcased remarkable stability, as evidenced in Figure 19b,c.

### 4.4. Electronic Structure

**Figure 20:**
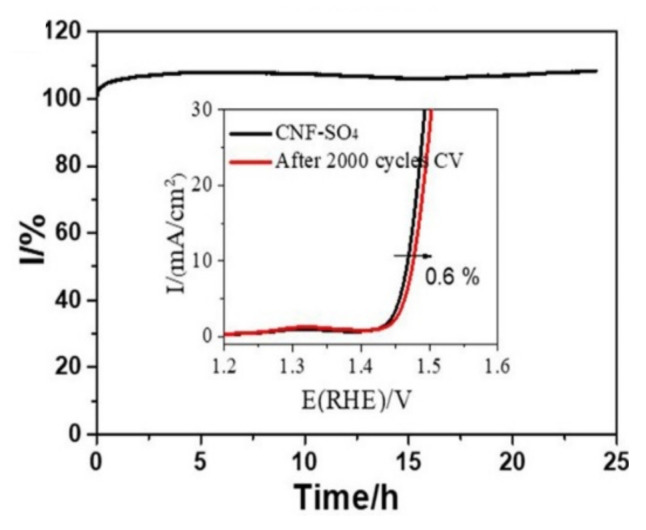
Stability tests by ChronoAmperometry at 1.461 V vs. RHE and 2000 cycles CV method for CNFSO_4_. Adapted with permission. Copyright 2020, Elsevier [133].

Maintaining precise control over the electronic structure is vital for augmenting the stability of acidic OER catalysts. Many materials, when exposed to the electrochemical conditions of acidic OER, are prone to rapid oxidation, resulting in significant deactivation. Thus, the inclusion of electron-donating components can shield active sites from over-oxidation, mitigating swift declines in catalytic efficacy [130,131,132]. Fine-tuning the electronic structure at these active sites can enhance intermediate adsorption, thus amplifying the kinetics of the oxygen evolution reaction (OER). In a pertinent study, Zhang et al. [133] incorporated solid acid structures into the electrocatalyst by tethering SO_4_^2−^ onto the surface of CoNiFeO_x_. DFT analyses suggested that the core of this solid acid modification was the emergence of Co_4_^+^ and intermediate spin Co^3+^(t2g5eg1), subsequently elevating the energy tiers of both the metal d-band and the oxygen p-band. This SO_4_^2−^ enhanced CNF-SO_4_ showcased exemplary electrochemical prowess, necessitating an overpotential of just 231 mV at 10 mA·cm^−2^ and a Tafel slope of merely 33.8 mV·dec^−1^. Notably, at 1.461 V vs. RHE (refer to Figure 20), CNF-SO_4_ sustained impressive stability spanning 24 h.

### 4.5. Electrochemical Electrode Modification

To bolster the stability of OER catalysts, Daniel Escalera-Lopez and his team introduced a novel approach centered on electrochemical electrode modification [134]. They augmented the OER resilience of IrO_x_ films under acidic environments by electrochemically decomposing a WS_4_^2−^ aqueous precursor and subsequently adapting the IrO_x_ film with amorphous tungsten sulfide (WS_3−x_). This method hinges on the pulsed electrodeposition technique, wherein the iridium film’s surface undergoes modification with transition metal oxides.

**Figure 21:**
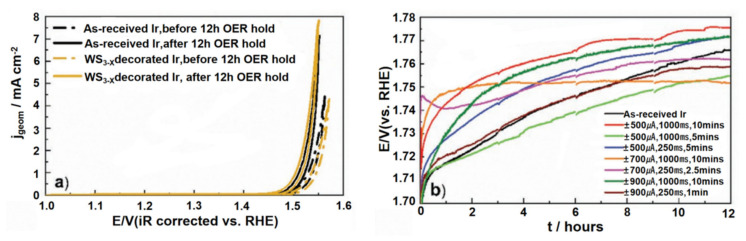
CVs. obtained from pristine (black) and WS_3−x_ embellished (±500 μA, 1000 ms, 5 min, dark yellow) Si/Cr/Ir electrodes: (**a**) prior to (dashed line) and following (continuous line) extended OER stability tests, with a sustained jgeom of 10 mA cm^−2^ over 12 h. The voltage span is set between 1.0 and 1.6 V versus RHE, and the scan rate at 10 mV s^−1^; (**b**) initial OER voltammograms captured for both the unaltered and WS_3−x_ adorned electrodes (±700 μA, 250 ms, 10 min) in a 0.1 m HClO_4_ electrolyte. Adapted with permission. Copyright, 2021, John Wiley and sons [134].

The OER robustness of both the unaltered materials and the WS_3−x_ tailored Ir specimens was assessed via linear voltammetry. Over a span of 12 h, chronopotentiometric charts were captured to track voltage alterations necessary to sustain an anodic current density of 10 mA cm^−2^, as illustrated in Figure 21b. After this 12 h OER assessment, it was discerned that both the geometric current density and mass activity were on par or even fell below those of the pristine Ir electrode. This implies that the OER efficacy of the WS_3−x_ amended Ir electrode is constricted by the passivation of its nuclei, as highlighted in Figure 21a. Yet, a distinct uptick in Rf was detected, offering a deeper perspective into the OER stability. Regardless of the OER test’s span, be it short-lived or protracted, the WS_3-x_ tailored Ir specimens manifested heightened OER durability and an amplified specific activity.

### 4.6. Other Factors to Consider in Improving the Activity and Stability of Acidic Water Electrolysis Catalysts

Although the water oxidation mechanism follows the LOM or AEM route under different pH conditions, the catalyst needs better corrosion resistance and solubility resistance under strong acid conditions. Therefore, in order to achieve higher activity and stability under acidic conditions, the following factors need to be additionally considered [135]:

Improving the thermal stability of the catalysts: A direct solution to solve catalyst instability is to use materials with a wider stability window, such as metal oxides as catalysts, with the main goal being to obtain catalysts with lower M_aq_ equilibrium concentrations at the same OER potential. To implement this strategy, it is often necessary to combine Pourbaix diagrams and a high-throughput screening method for analysis. Wang et al. [136] identified 68 likely acid-stable non-noble metal candidates using a high-throughput screening method, and the stability of at least one family of these oxide candidates, namely Mn-Sb, has been experimentally verified.

Protecting unstable interfaces with overlayers: By constructing a loose and porous physical protective layer on the surface of the catalyst through electrodeposition or chemical deposition, on the one hand, it can ensure that the electrocatalyst does not come into contact with the electrolyte and serve as a diffusion barrier for dissolved substances that affect the dynamic balance. On the other hand, it can electronically enhance cation stability in the oxide lattice. Common stable, protective layers in acid media include titanium dioxide, carbon materials, etc. For example, using atomic layer deposition, Tran-Phu et al. [137] revealed that Co_3_O_4_ covered with 4.4 nm of amorphous TiO_2_ delivers the best performance, with a ca. 3-fold increase in the lifetime compared to unprotected Co_3_O_4_. It should be noted that the thickness control of the layer is crucial. If it is too thick, it will affect the activity of the catalyst, and if it is too thin, it will not have any protective effect.

Suppressing dissolution kinetics: The dissolution problem of the catalyst will exist under both acidic and alkaline conditions. This effect is more obvious under acidic conditions, especially under long-term operating conditions. It can be mainly divided into two major categories of factors, including the intrinsic dissolution of the catalyst itself (such as surface reconstruction and active site dissolution) and external instability factors (such as substrate passivation and catalyst shedding). However, there is currently no good design strategy to deal with this challenge, and the corresponding theory is lacking. The usual idea is to adjust the electronic structure of the catalyst to inhibit its dissolution reaction, but this often reduces its catalytic activity; another idea is the alloying method. Developing long-term, stable catalysts still requires extensive experimentation.

## 5. Summary and Prospect

The scale of development of water spitting in acid depends largely on the development of electrocatalysts. The preparation of OER catalysts in acid with good stability and activity still faces many challenges.

In this paper, the mechanism of OER is analyzed, and the characteristics of the adsorbate evolution mechanism (AEM) and lattice oxygen oxidation mechanism (LOM) are summarized. AEM tends to be a more stable but less active oxidation process, and LOM exhibits excellent activity but is prone to catalyst degradation. Then, in order to improve the activity of the catalysts, strategies were summarized and analyzed in terms of composition design, morphology design, and electronic structure, respectively. The stability of the catalyst can be improved by optimizing the structural composition, reducing lattice oxygen oxidation, electron coupling, electronic structure, and electrochemically modified electrodes.

In the future, more stable and active acids, such as OER catalysts in acid, will surely appear, which is also pivotal to the progress of science and technology. However, the route to finding better catalysts is bound to be full of twists and turns, and it requires a combination of advanced characterization techniques, theoretical calculations, and specific experiments to gain a deeper understanding of the mechanism and reaction pathways of acid OER catalysts in acid. Finally, it is hoped that this review can provide some assistance in the design and development of highly stable and active OER catalysts under acidic conditions.

## Figures and Tables

**Table 1:** Performance of OER non-noble metal electrocatalysts in acidic medium.

Catalyst	Overpotential 10 mA cm^−2^	Tafel Slope	Stability	Mass Activity 300 mV	Ref.
TiO_2_-modified MnO_2_	510 @ 1 mA cm^−2^	170	2 h @ 1.8 V vs. RHE	-	[22]
F-doped Cu_1.5_Mn_1.5_O_4_	320 @ 9.15 mA cm^−2^	60	24 h @ 1.55 V vs. RHE	61 A g^−1^ @ 320 mV	[23]
C_3_N_4_–CNT	370	129	14 h @ 1.63 V vs. RHE		[24]
1 T MoS_2_	420	322	2 h @ 10 mA cm^−2^	5 A g^−1^	[25]
Co_3_O_4_-CP	370	82	86.8 h @ 100 mA cm−2		[26]
Co_3_O_4_	570		12 h @ 10 mA cm^−2^		[27]
Ni_0.5_Mn_0.5_Sb_1.7_O_y_	672	85	168 h @ 10 mA cm^−2^		[28]
Ba [Co-POM]	361	97	24 h @ 1.48 V		[29]
N-WC nanoarray	250		1 h @ 10 mA cm^−2^		[30]
Ni_2_Ta	570		66 h @ 10 mA cm^−2^		[31]
Ni_42_Li_2_O_5_	445	260	41.7 h @ 10 mA cm^−2^		[32]
Co-doped MoS_2_ nanosheets	670	245.92	11.11 h @ 1.8 V vs. RHE		[33]
Co_2_TiO_4_	513	320			[34]
TiB_2_	560		11 h @ 10 mA cm^−2^		[35]
Co_0.05_Fe_0.95_O_y_	650	110	50 h @ 10 mA cm^−2^		[36]
γ-MnO_2_	428	80	8000 h @ 10 mA cm^−2^		[37]
Co-ZIF	405	281	12 h @ 1.635 V vs. RHE		[38]
CoSAs-MoS_2_/TiN NRs	454.9 mV	165.5	45 h @ 50 mA cm^−2^		[39]
MnFeF_4.6_O_0.2_		195	20 h @ 10 mA cm^−2^		[40]

**Table 2:** Summary of electrochemical activities of Rh_x_Ir_(100−x)_/VXC catalysts with different compositions.

Catalyst	η @ 10 mA cm^−2^ (mV)	m @ 1.53 V vs. RHE (A mg^−1^ Metal)	j_m_ @ 1.53 V vs. RHE (A mg^−1^ Ir)	TOF (s^−1^)	Tafel Slope (mV dec^−1^)
Rh/VXC	495 ± 9	0.060 ± 0.006	-	0.371 ± 0.040	141
Rh_73_Ir_27_/VXC	371 ± 7	0.172 ± 0.021	0.422 ± 0.050	0.993 ± 0.119	108
Rh_49_Ir_51_/VXC	314 ± 2	0.482 ± 0.013	0.730 ± 0.020	3.166 ± 0.085	103
Rh_22_Ir_78_/VXC	292 ± 1	1.020 ± 0.017	1.174 ± 0.020	5.095 ± 0.085	101
Ir/VXC	340 ± 11	0.349 ± 0.051	0.349 ± 0.051	1.745 ± 0.256	111

**Table 3:** Summary of ECSA, TOF of Rh_x_Ir_(100−x)_/VXC catalysts with different compositions.

Catalyst	ECSA (cm^2^)	TOF_ECSA_(s^−1^)	TOF_TEM_ (s^−1^)
Rh/VXC	495 ± 9	0.371 ± 0.040	0.072 ± 0.008
Rh_73_Ir_27_/VXC	371 ± 7	0.993 ± 0.119	0.200 ± 0.024
Rh_49_Ir_51_/VXC	314 ± 2	3.166 ± 0.085	0.533 ± 0.014
Rh_22_Ir_78_/VXC	292 ± 1	5.095 ± 0.085	1.015 ± 0.017
Ir/VXC	340 ± 11	1.745 ± 0.256	0.278 ± 0.041

## Data Availability

Data are contained within the article.
